# The brain’s structural differences between postherpetic neuralgia and lower back pain

**DOI:** 10.1038/s41598-021-01915-x

**Published:** 2021-11-17

**Authors:** Jianxing Qiu, Mengjiao Du, Junzhe Yang, Zengmao Lin, Naishan Qin, Xiaowei Sun, Linling Li, Rushi Zou, Juan Wei, Bing Wu, Jing Liu, Zhiguo Zhang

**Affiliations:** 1 Department of Radiology, Peking University First Hospital, 8 XiShiKu Avenue, XiCheng District, Beijing, 100034 China; 2 School of Biomedical Engineering, Health Science Center, Shenzhen University, Shenzhen, China; 3 Department of Anesthesiology, Peking University First Hospital, Beijing, China; 4 GE Healthcare China, Beijing, China; 5 Guangdong Provincial Key Laboratory of Biomedical Measurements and Ultrasound Imaging, Shenzhen University, Shenzhen, China; 6 Marshall Laboratory of Biomedical Engineering, Shenzhen University, Shenzhen, China; 7 Peng Cheng Laboratory, Shenzhen, China

**Keywords:** Neural circuits, Neuronal physiology

## Abstract

The purpose is to explore the brain’s structural difference in local morphology and between-region networks between two types of peripheral neuropathic pain (PNP): postherpetic neuralgia (PHN) and lower back pain (LBP). A total of 54 participants including 38 LBP and 16 PHN patients were enrolled. The average pain scores were 7.6 and 7.5 for LBP and PHN. High-resolution structural T1 weighted images were obtained. Both grey matter volume (GMV) and morphological connectivity (MC) were extracted. An independent two-sample t-test with false discovery rate (FDR) correction was used to identify the brain regions where LBP and PHN patients showed significant GMV difference. Next, we explored the differences of MC network between LBP and PHN patients and detected the group differences in network properties by using the two-sample t-test and FDR correction. Compared with PHN, LBP patients had significantly larger GMV in temporal gyrus, insula and fusiform gyrus (*p* < 0.05). The LBP cohort had significantly stronger MC in the connection between right precuneus and left opercular part of inferior frontal gyrus (*p* < 0.05). LBP patients had significantly stronger degree in left anterior cingulate gyrus and left rectus gyrus (*p* < 0.05) while had significantly weaker degree than PHN patients in left orbital part of middle frontal gyrus, left supplementary motor area and left superior parietal lobule (*p* < 0.05). LBP and PHN patients had significant differences in the brain’s GMV, MC, and network properties, which implies that different PNPs have different neural mechanisms concerning pain modulation.

## Introduction

Increasing incidence of chronic pain has drawn more and more attention worldwide. It was demonstrated that chronic pain could alter brain activity and networks^4,13,14,42,47^. Moreover, different types of chronic pain might alter different brain regions and networks, which was called brain biomarkers or pain matrix^25,33,36,49^. Investigation on brain network alteration have benefits for revealing central modulation mechanism of pain as well as for further research of precise and targeted therapy^9,21,38^.

As a common type of chronic pain, peripheral neuropathic pain (PNP)^17,40^ has typic characteristics of spontaneous pain due to inflammation, such as postherpetic neuralgia (PHN)^20,22^, or due to injury of nerve roots, such as lower back pain (LBP) caused by compression of lumbar discs^3,55^. Both PHN and LBP may have the same pain mechanism and clinical symptoms, but they still induce different central activity and pain biomarkers. A large number of neuroimaging studies have explored the brain activity and network changes^16,27,28,30,46^ in PHN and LBP, and have exposed their neural mechanism of pain modulation. However, most previous studies have conducted research on pain patients with healthy subjects as control^27,30^, or used self-control before and after therapy^16,27^. As we know, pain disorders could bring pain feeling and some negative emotion such as depression, insomnia and anxiety, which would also alter the brain’s structure and functions^31,33,49^. Therefore, it remains unclear whether previous findings of altered brain patterns in pain patients were caused by pain or accompanying negative emotion. To solve this problem, using two types of pain diseases as control for each other could help exclude emotional influence and facilitate exploring specific brain regions or networks for different types of pain. Pain patients always suffer from negative emotions, it is helpful to offset the effects by enrolling patients with comparable pain intensities and emotion scores by questionnaires as control. In addition, making PHN and LBP as controls for each other might be more possible to obtain the specific brain biomarker for different pain even though both of them belong to PNP. To our best of knowledge, there was no neuroimaging study concerning both PHN and LBP.

Multimodal MRI is the most commonly used neuroimaging method in previous pain-related studies^16,27,28,30,46^, and it can reveal the functions and structures of the brain from different aspects. Compared with function MRI (fMRI) and diffusion tenson imaging (DTI), T1 weighted imaging (T1WI) has the advantages of easier access, higher resolution, and relative insensitivity to artifacts (e.g., head motion, susceptibility). However, few studies^12,29^ have explored the GMV changes in PHN from T1WI, let alone MC.

By calculating the inter-regional similarities of local brain morphology from T1WI^7,24,43^, MC could help characterize structural connectivity and reflect synchronized development^2,15^. Previous studies have shown MC could be important brain biomarkers of perception^26^, neurological diseases^52^, and pain sensitivity^58^. However, MC has not been conducted in pain patients. Considering the mounting evidence on the close relationship between pain disorders and other types of structural or functional brain connectivity (DTI tractography or functional connectivity)^2,35^, it is reasonable to hypothesize that MC is a promising brain biomarker of neuropathic pain.

In our study, we explored both the GMV and MC patterns of PHN and LBP patients and gauged the differences of structural networks between these two types of PNP to find out the possible structural MRI biomarkers which were more specific to the diseases. Because PHN and LBP have different causes, we hypothesized that PHN and LBP would have different structural networks even if they both belong to PNP.

## Material and methods

### Ethics statement

This prospective study was approved by an institutional review board (Peking University First Hospital Ethics committment) and was conducted in compliance with the Declaration of Helsinki. All participants in this study provided written informed consent.

### Participants

Consecutive patients which were confirmed as PHN or LBP by anesthesiologists were included. All the subjects were right-handed. For PHN cohort, all participants reported a history of shingles, associated pain, and varicella zoster virus infection. All participants reported a history of persistent pain for at least 2 months after resolution of the acute shingles episode. For LBP cohort, all participants were suffering from chornic lower back pain for at least 6 months according to IASP criteria^32^. Pain was primary localized within the lumbar or lumbosacral region, accompanying with or without radiation to the buttocks, thighs or legs. In addition, all the LBP patients also underwent lumbar MR scanning to identify the herniation of lumbar discs.

None of them had a history of psychiatric or neurological disorders. Individuals with a history of any disorder with a potential impact on brain structure (such as hypertension requiring medical treatment, traumatic brain injury, diabetes mellitus, rheumatologic disorders, and any other chronic pain disease different from PHN and LBP) were excluded from this study. The clinical symptoms of pain were assessed using a numerical rating scales (NRS), with a range from 0 (no pain) to 10 (the highest tolerable pain). All of the patients underwent neurological and psychological examinations and fulfilled the mini-mental state examination (MMSE). Only the patients with the scores above 27 in MMSE were included. Patients with any abnormality detected on brain MRI were excluded from our study.

For further characterization of patients, all individuals were asked to complete the Chinese version of the Hospital Anxiety and Depression Scale (HADS)^57^ for measuring anxiety and depression.

### Data acquisition and preprocessing

All the brain MRI examinations were performed on a 3.0 T scanner (GE healthcare, 750HD) with a 32-channel phased-array head coil. High-resolution structural T1WI were obtained by using a three-dimensional magnetization-prepared rapid gradient-echo (3D-MPRAGE) sequence with the following parameters: repetition time (TR) = 8.1 ms; echo time (TE) = 3.7 ms, flip angle = 8^0^; slice thickness = 1 mm without a gap; field of view (FOV) = 240 × 240 mm^2^; matrix size = 256 × 256. A total of 160 axial slices were acquired for each patient.

All structural MRI data processing routines were carried out by using the Statistical Parametric Mapping 12 (SPM12, https://www.fil.ion.ucl.ac.uk/spm/software/spm12/) Toolbox in MATLAB-R2018b (The MathWork, Inc., Natick, MA, US). The preprocessing procedure was as follows. First, the MRI data of each subject were segmented into grey matter (GM), WM and cerebrospinal fluid (CSF). Second, the GM segments were non-linearly co-registered by using the inbuilt high dimensional Diffeomorphic Anatomical Registration Through Exponentiated Lie Algebra (DARTEL)^5^. Third, GM images were normalized to standard Montreal Neurological Institute (MNI) space to make the GM images in the same space. Thereafter, the resulting GM images were modulated by the Jacobian determinants. Fourth, the GM images were smoothed with an 8 mm full-width-half-maximum (FWHM) Gaussian kernel. The GM images of all subjects were used for the further analysis.

We used the Automated Anatomical Labeling (AAL) atlas to define the whole brain parcellation for later MC network analysis^45^. The cerebellar regions were excluded due to incomplete coverage of the cerebellum in several participants. Thus, a total of 90 brain regions of interest (ROIs) were defined in this analysis.

### GMV and MC estimation

In our study, we extracted two types of features using the preprocessed GM images: GMV and MC. GMV of each subject was estimated by voxel-based morphometry (VBM) in the whole brain as the local morphological feature. MC is a measure of structural connectivity, and it was calculated in the following steps. First, we quantified the GM intensity of each voxel within each ROI in preprocessed GM images. Second, the kernel density estimation with automatically chosen bandwidths^10^ was used to calculate the probability density function for each ROI^11^. Third, the morphological connectivity for each pair of ROIs was estimated as the similarity between the two probability density functions of this pair of ROIs by using Kullback–Leibler divergence^23^. The Kullback–Leibler divergence was defined as:

1 $$KL\left(p,q\right)={\int }_{X}\left(p\left(x\right)\mathrm{log}\left(\frac{p\left(x\right)}{q\left(x\right)}\right)+q(x)\mathrm{log}\left(\frac{q(x)}{p(x)}\right)\right)$$

where $p\left(x\right)$ and $q(x)$ were the probability density functions of two ROIs $p$ and $q$, respectively.

Moreover, Kullback–Leibler divergence was converted to a similarity metric as^24^:

2 $$KLS\left(p,q\right)={e}^{-KL(p,q)}$$

The range of Kullback–Leibler-based similarity (KLS) is from 0 to 1, where 1 indicates two identical distributions while 0 implies two completely different distributions. Finally, a MC matrix with a size of 90 × 90 was acquired for each participant.

### Graph-theoretical network analysis

Next, we applied graph theory to estimate the network properties of MC, and the network properties including degree, small-worldness, network efficiency, clustering coefficient and characteristic path length. The calculation of network properties on MC matrices across all participants were performed with the GRETNA Toolbox^51^. First of all, a thresholding procedure is commonly used to binarize the MC network before performing topological characterization on the morphological connectivity matrices. A sparsity threshold, which was defined as the ratio of the number of existing edges divided by the maximum possible number of edges in a network, was used to binarize the divided MC network^23^. We binarized the MC network within a wide range of the sparsity threshold (from 0.05 to 0.4 with an interval of 0.02) because an automated method to determine the sparsity threshold is lacking^1,50^. Then, we calculated the network properties of MC within different sparsity thresholds.

### Statistical analysis

Independent-*t*-test was used for the comparisons of age and scores for pain, anxiety and depression between groups.

An independent two-sample t-test with an accompanying false discovery rate (FDR) correction was used to identify the brain regions where LBP and PHN patients showed significant GMV difference. Meanwhile, the total intracranial volume (TIV) of each subject was estimated and used as a covariate to remove the effect of variations in brain intracranial volume. Next, in order to explore the differences of MC network between LBP and PHN patients, we performed an independent two-sample t-test with FDR correction to correct the problem of multiple comparisons^8^. Furthermore, we detected the group differences in network properties (including degree, small-worldness, network efficiency, clustering coefficient, and characteristic path length) at different sparsity thresholds by using the two-sample t-test and FDR correction.

### Correlations analysis

Next, for each the feature showing significant group differences, we calculated the Pearson’s correlation coefficients between these features (including MC and degree) and pain scores in LBP or PHN group, separately. On the other hand, the standard general linear model (GLM) was used by creating design matrices for multiple regression analysis of GMV, with pain scores and TIV as regressors. The GLM was used to construct pseudo t statistic images, and significant clusters were extracted with voxel-level p < 0.005. FDR was finally used to correct the problem of multiple comparisons.

### Ethical approval

Ethical approval was obtained from the institutional review board (Peking University First Hospital Ethics committment).

### Consent to participate

All participants in this study provided written informed consent.

## Results

### Clinical characteristics

A total of 54 participants including 38 LBP patients (11 males, 27 females, age: 59 ± 12) and 16 PHN patients (3 males, 13 females, age: 66 ± 7) were enrolled in the experiment. There was no statistical significance between age comparison (*t* = − 1.852, *p* = 0.07 > 0.05).

The average pain scores were 7.6 ± 1.6 (rang from 5.5 to 10) in LBP patients, and were 7.5 ± 1.9 (rang from 6 to 10) in PHN patients. There was no statistical significance between them (*t* = 0.111, *p* = 0.912 > 0.05).

There were also no statistical significance in pain duration, scores for psychological state, and the details of demographic and clinical characteristics were in Table 1.

**Table 1:** Demographic and clinical characteristics of the participants.

	LBP (n = 38)	PHN (n = 16)	*t*	*p*
Age (y)	59 ± 12	66 ± 7	− 1.852	0.07
Pain scores	7.6 ± 1.6	7.5 ± 1.9	0.111	0.912
Pain duration (months)	40 ± 46	22 ± 30	1.665	0.103
Scores for anxiety	9.7 ± 4.6	10.6 ± 5.2	− 0.669	0.507
Scores for depression	11.1 ± 4.9	10.7 ± 5.7	0.272	0.786

### Group differences in GMV

**Figure 1:**
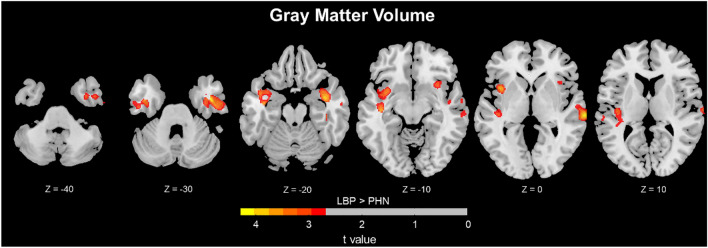
Difference in GMV between LBP and PHN. (LBP > PHN, P < 0.05, Cluster-level FDR corrected.). GMV differences observed when comparing LBP and PHN patients. After controlling for total intracranial volume, Increased GMV in LBP was observed in several brain regions, ie., the temporal gyrus, fusiform gyrus and Insula (*p* < 0.05, Cluster-level FDR corrected).

The clusters with significant differences in GMV between LBP and PHN were shown in Table 2 and Fig. 1. Compared with PHN, LBP patients had significantly larger GMV in temporal gyrus, insula and fusiform gyrus (*p* < 0.05, cluster-level FDR-corrected).

**Table 2:** Difference in GMV between LBP and PHN.

No. Cluster	Cluster size	MNI			Peak t value	p value	Brain regions
		x	y	z			
1	3544	42	− 2	− 32	4.27	0.001	Temporal_Inf_R Temporal_Sup_R Temporal_Mid_R Fusiform_R Insula_R Temporal_Pole_Sup_R
2	2796	− 41	− 14	− 15	3.91	0.002	Temporal_Sup_L Insula_L Temporal_Inf_L Heschl_L Temporal_Pole_Sup_L

*Sup* superior, *Mid* middle, *Inf* inferior.

### Group differences in MC

**Figure 2:**
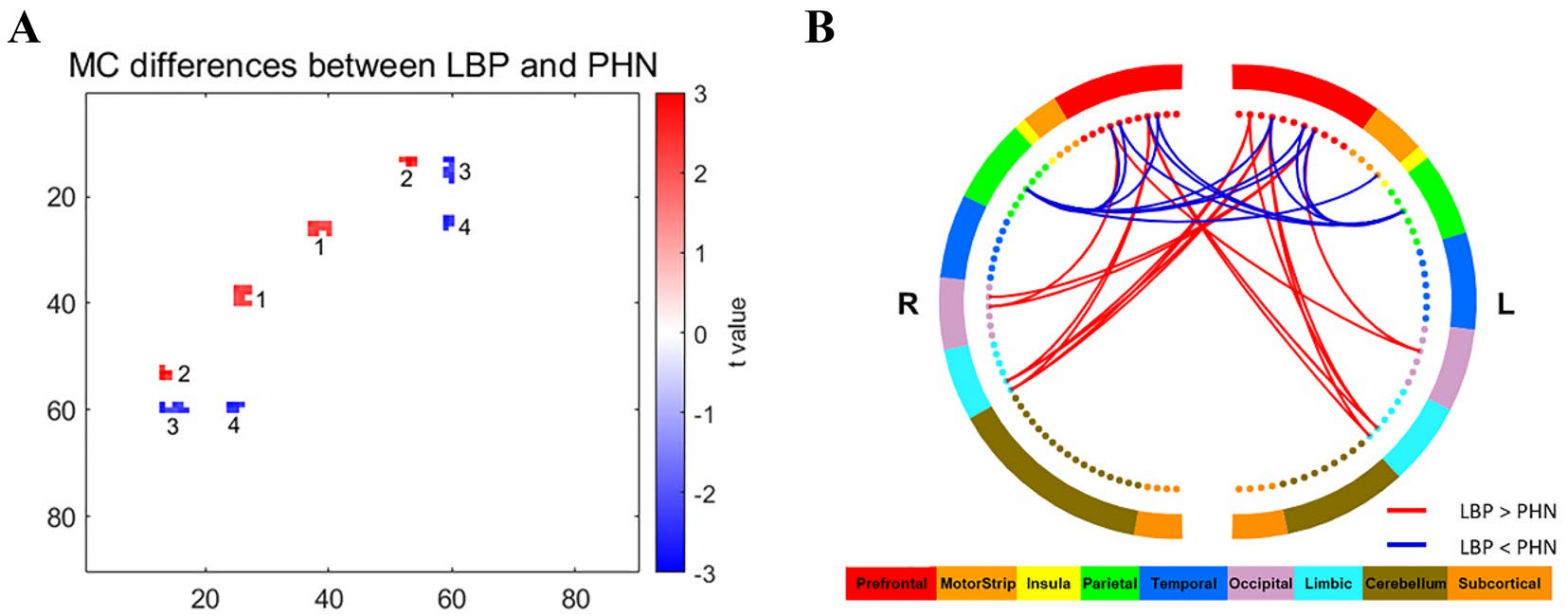
Group differences in MC network between LBP and PHN. (**A**) Group differences in MC network between LBP and PHN. Increased MC in LBP patients were observed in red regions, i.e., cluster 1 and cluster 2 (p < 0.05). On the other hand, decreased MC in LBP patients were observed in blue regions, i.e., cluster 3 and cluster 4 (p < 0.05). Color bar represents t values. (**B**) Clusters with different MC in LBP compared with PHN. Red lines represent increased MC in LBP, while blue lines represent decreased MC in LBP. Color bar represents different brain regions.

Group differences in MC network between LBP and PHN were shown in Fig. 2, in which the MC differences appeared as a set of clusters in small areas. We only considered a cluster with more than 4 MCs, so a total of 4 clusters were observed. Detailed information of the 4 clusters was shown in Table 3.

**Table 3:** Clusters with significant different MC between LBP and PHN.

Cluster	Region-pairs of brain	*t* value	*p* value ^a^
1	Hippocampus_L—Frontal_Med_Orb_L	2.3403	0.0231
1	Hippocampus_R—Frontal_Med_Orb_L	2.1732	0.0343
1	ParaHippocampal_L—Frontal_Med_Orb_L	2.0144	0.0491
1	ParaHippocampal_R—Frontal_Med_Orb_L	2.0993	0.0407
1	Hippocampus_L—Frontal_Med_Orb_R	2.0634	0.0441
1	Hippocampus_R—Frontal_Med_Orb_R	2.2518	0.0286
1	ParaHippocampal_L—Frontal_Med_Orb_R	2.2095	0.0316
1	ParaHippocampal_R—Frontal_Med_Orb_R	2.1778	0.0340
1	Hippocampus_L—Rectus_L	2.1966	0.0325
1	Hippocampus_R—Rectus_L	2.3418	0.0231
1	ParaHippocampal_R—Rectus_L	2.1585	0.0355
2	Occipital_Mid_R—Frontal_Inf_Tri_L	2.2629	0.0278
2	Occipital_Inf_L—Frontal_Inf_Tri_L	3.0399	0.0037
2	Occipital_Inf_R—Frontal_Inf_Tri_L	2.6729	0.0100
2	Occipital_Inf_L—Frontal_Inf_Tri_R	2.8535	0.0062
2	Occipital_Inf_R—Frontal_Inf_Tri_R	2.1962	0.0326
3	Parietal_Sup_L—Frontal_Inf_Tri_L	− 2.5317	0.0144
3	Parietal_Sup_R—Frontal_Inf_Tri_L	− 2.5476	0.0138
3	Parietal_Sup_R—Frontal_Inf_Tri_R	− 2.0434	0.0461
3	Parietal_Sup_L—Frontal_Inf_Orb_L	− 2.3819	0.0209
3	Parietal_Sup_R—Frontal_Inf_Orb_L	− 2.0119	0.0494
3	Parietal_Sup_L—Frontal_Inf_Orb_R	− 2.1010	0.0405
3	Parietal_Sup_R—Frontal_Inf_Orb_R	− 2.3625	0.0219
3	Parietal_Sup_R—Rolandic_Oper_L	− 2.4294	0.0186
4	Parietal_Sup_L—Frontal_Sup_Medial_R	− 2.6469	0.0107
4	Parietal_Sup_R—Frontal_Sup_Medial_R	− 2.2846	0.0264
4	Parietal_Sup_L—Frontal_Mid_Orb_L	− 2.5650	0.0132
4	Parietal_Sup_R—Frontal_Mid_Orb_L	− 2.2882	0.0262
4	Parietal_Sup_L—Frontal_Mid_Orb_R	− 2.3211	0.0242

^a^independent two-sample t-test.

**Figure 3:**
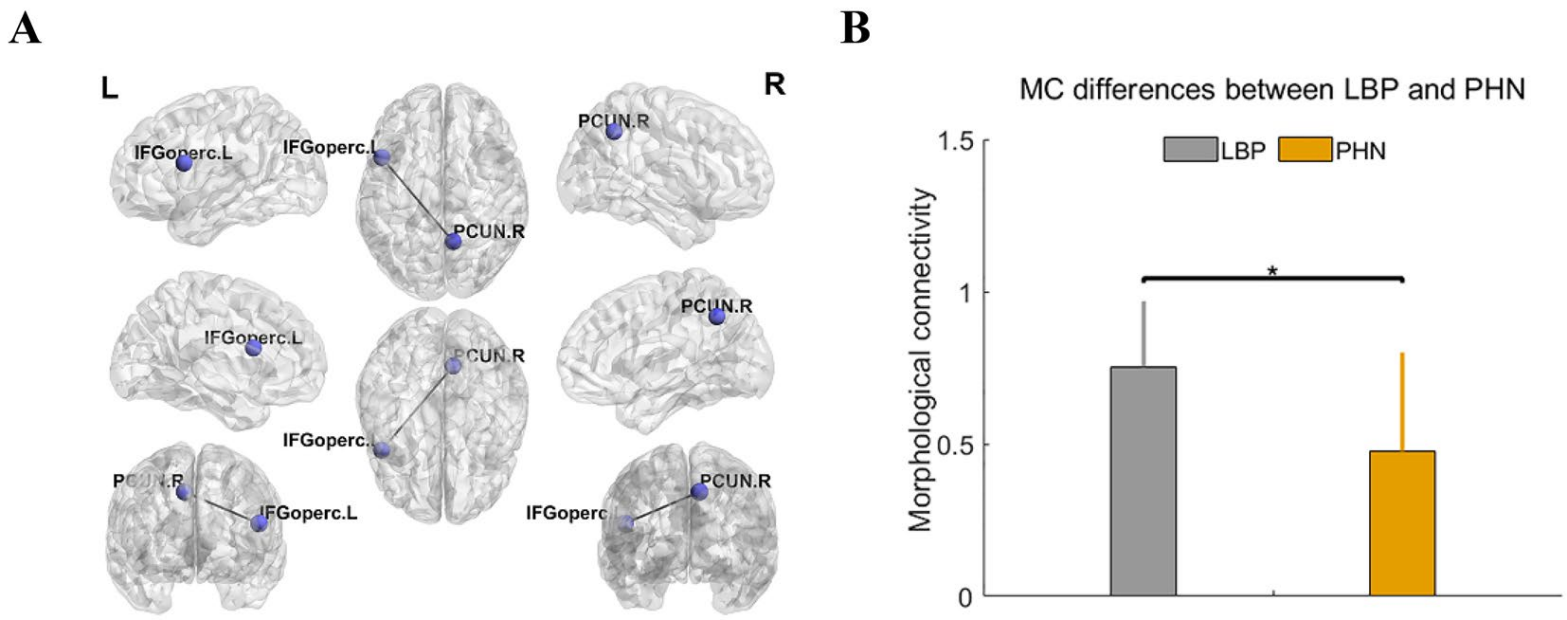
Group differences in MC between LBP and PHN after FDR correction. (**A**) Group differences in MC between LBP and PHN after FDR correction. Significantly increased MC in LBP (p < 0.05, FDR) were observed between right precuneus (PCUN. R) and left opercular part of inferior frontal gyrus (IFGoperc. L). (**B**) Bar plots showing the group difference in MC between LBP and PHN. * indicated p < 0.05, FDR corrected.

Compared with the PHN cohort, the LBP cohort had significantly stronger MC in cluster 1 (including hippocampus and orbital gyrus) and cluster 2 (including inferior occipital gyrus and inferior frontal gyrus of triangle) (*p* < 0.05), and had significantly weaker MC in cluster 3 (including superior parietal gyrus and orbital gyrus) and cluster 4 (including superior parietal gyrus, medial superior frontal gyrus and orbital gyrus) (*p* < 0.05). However, after FDR correction, we only found that the LBP cohort had significantly stronger MC than PHN in the connection between right precuneus (PCUN. R) and left opercular part of inferior frontal gyrus (IFGoperc. L) (*p* < 0.05, FDR), and the results were shown in Fig. 3.

### MC network properties: differences in degree

**Figure 4:**
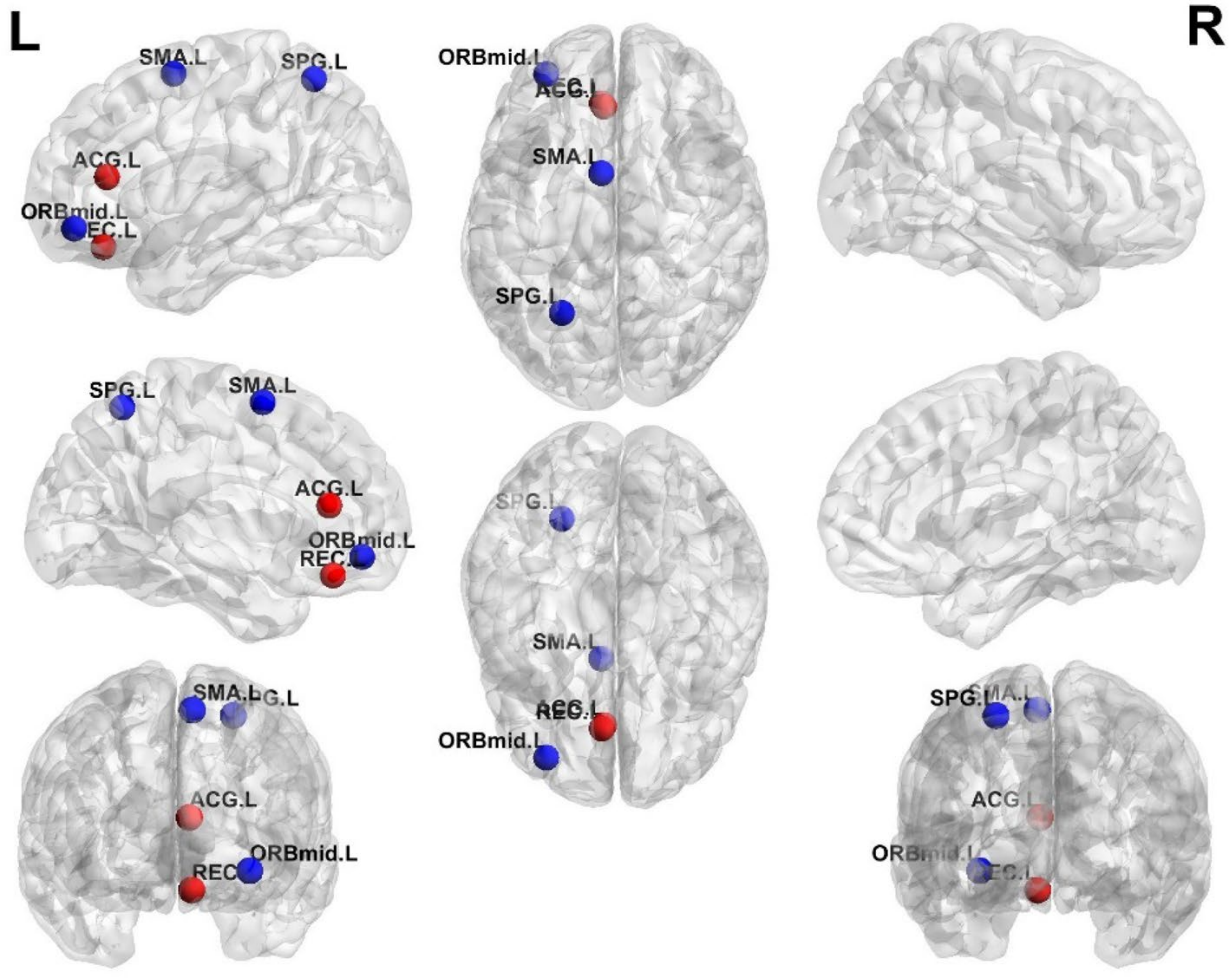
Group differences in degree between LBP and PHN within different sparsity. Significantly increased degree in LBP patients were observed in red regions, including left ACG and left REC (*p* < 0.05, FDR). Significantly decreased degree in LBP were observed in blue regions, including left SMA, left SPG and left middle ORB (*p* < 0.05, FDR).

The group differences in degree between LBP and PHN patients were shown in Fig. 4. LBP patients had significantly stronger degree than PHN patients in left anterior cingulate gyrus (ACG. L) and left rectus gyrus (REC. L) (*p* < 0.05, FDR), while LBP patients had significantly weaker degree than PHN patients in left orbital part of middle frontal gyrus (ORBmid. L), left supplementary motor area (SMA. L) and left superior parietal lobule (SPG. L) (*p* < 0.05, FDR). In addition, there were no significant differences in other network properties (small-worldness, network efficiency, clustering coefficient and characteristic path length) between LBP and PHN patients.

### Correlations analysis

**Figure 5:**
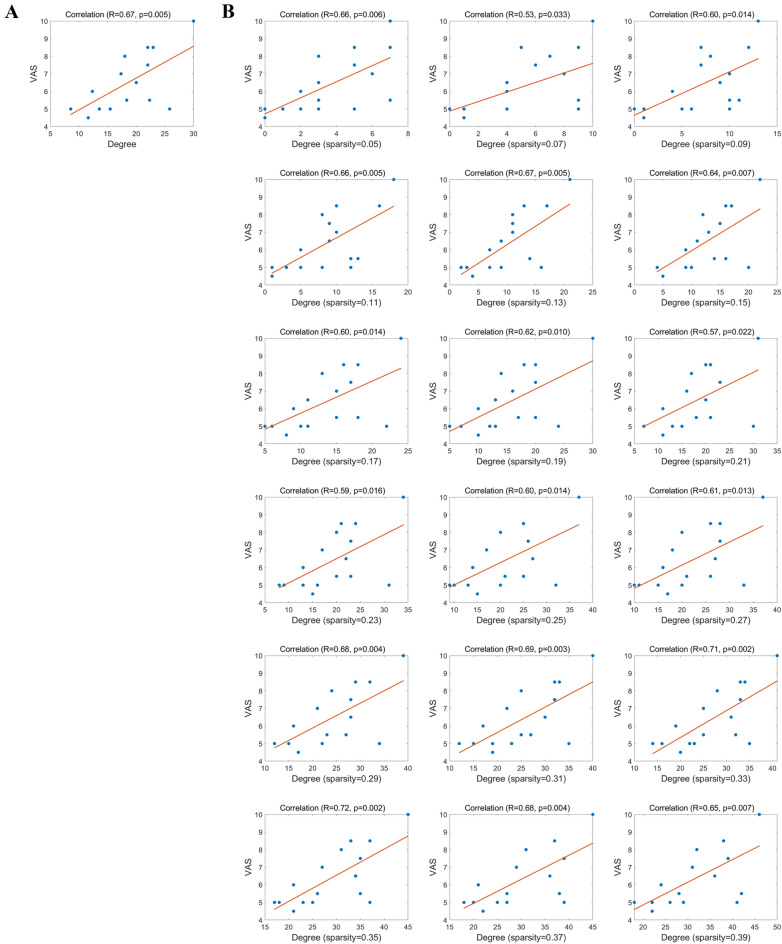
Correlations between pain scores and degree in PHN within different sparsity. (**A**) Correlations between pain score and mean degree of the left rectus gyrus (REC.L) in PHN patients. The mean degree of the REC.L was significantly positively correlated with the pain score (R = 0.67, p = 0.005). (**B**) Correlations between pain score and mean degree of the REC.L in PHN patients within different sparsity.

The correlations between pain scores and the features that showing significant group differences were shown in Fig. 5. We found that the mean degree of left rectus gyrus in PHN patients was significantly positively correlated with the pain scores (R = 0.67, p = 0.005, FDR), and the degree of left rectus gyrus in PHN patients within different sparsity were positively correlated with the pain scores (see Fig. 5B). However, there were no significantly correlations between MC or GMV and pain scores in PHN patients, and no correlations between these different features (including MC, degree and GMV) and pain scores in LBP patients.

## Discussion

In our study, we investigated the brain’s regional characteristics (GMV) and the brain’s inter-regional structural connections (MC) of PHN and LBP. Our results revealed that GMV and MC are capable of detecting the differences of the brain structure between two types of pain disorders, and the structural differences might be the specific areas for pain modulation.

In recent years, the whole brain connectivity^16,27,28,30,46^ has been widely explored in pain research. It has been widely recognized that pain is modulated by brain networks which consist of a set of inter-connected brain regions, not only by the separate brain areas^28,56^. Brain connectivity analysis, such as small-world networks and WM tractography, has become popular in investigating pain-related changes. Previous studies have shown that sMRI-derived MC is a promising measure of individual-level structural connectivity to investigate cognition, perception, and neurological disorders^26,52,53^. Some studies used fMRI-derived functional connectivity in the prediction of individual pain thresholds^41,44^, while it was also found that MC is related to functional connectivity^37^. Therefore, it is reasonable to consider the use of MC in pain research. A recent study^58^ has also found that the predictive capability of MC was significantly higher than the regional morphological features like GMV in prediction of individual pain sensitivity.

For the number of MCs in cluster, the different MCs between LBP and PHN patients were scattered in the whole brain, while only a few clusters could be clearly observed. By counting the number of clusters with the same number of MCs, we found a cluster containing 5 MCs was an inflexion point of the number of clusters, which means that most clusters only had 1–4 MCs. Actually, the number of clusters with 1, 2, 3, 4, or ≥ 5 MCs was 92, 40, 10, 4, and 4, respectively. Therefore, to better illustrate the different MCs between two types of patients, we only discussed significant clusters with more than 4 MCs (i.e., the number of MCs ≥ 5).

As for the physiological principle of inter-regional MC, some researchers have proposed the axon tension theory^48^: anatomically connected brain regions are modulated by a mechanical force, resulting in similar morphological properties. This theory could also be applicable to pain patients: brain regions involving pain modulation showed similar morphological distribution. In our study, most of brain regions detected, including ACG, medial frontal gyrus, SMA and superior parietal lobule, have been demonstrated to have a relationship with pain in previous studies. Our results could further prove the capability of MC in detecting brain connectivity involving pain modulation in two types of PNP disorders (PHN and LBP).

Many studies^16,27,28,30,46^ have utilized various MRI techniques to reveal the brain’s structural and functional abnormalities of PHN and LBP from different perspectives. Previous two PHN studies^12,29^ have shown that reduced GMV was found in the bilateral insula, precentral gyrus and right middle frontal gyrus (MFG) while increased GMV was found in the bilateral thalamus, right PHG, lentiform nucleus of PHN patients, as compared with normal controls. There were also a number of sMRI studies with various results in investigation of LBP. Generally, LBP patients had larger GMV than healthy people in bilateral putamen and left posterior thalamus^39^, and in temporal lobes, S1, S2, M1^46^. On the other hand, PHN patients also showed greater GMV in both thalamus and basal ganglia^12,29^, which suggested that these two regions were the main parts involved in pain modulation of both PHN and LBP. That could be the explanation for why both the GBV and MC results of these two areas showed no significance in PHN-LBP comparison in our study. These two regions may play the same role in modulating either PHN or LBP. In the following, we will discuss some major regions and connections identified in the present study.

1. Temporal gyrus. Many regions in the temporal gyrus showed greater GMV for PHN than for LBP, as shown in our study. In previous studies with healthy controls, temporal gyrus showed no significant changes in PHN, while showed greater GMV in LBP^46^. The same results could be found for fusion gyrus^30^. In addition, temporal gyrus was reported to have a great relationship with hearing ability. Therefore, LBP might affect auditory regions’ activity while PHN might not.
2. Insula. Insula has been involved in modulating various types of pain diseases, including LBP and PHN. Previous studies showed that the GMV of insula was smaller in both PHN and LBP, as compared with healthy people^6,29^. However, from PHN-LBP comparison in our study, insula seemed to be affected more by PHN because the GMV of insula was larger in LBP than in PHN.
3. ACG. ACG, which is often regarded to have a close relationship with acute pain or visceral pain^19,34^, showed more MC patterns in LBP, as compared with PHN. In Luchtmann’s study^30^ with 12 LBP patients, GMV of ACG was larger than that of healthy people. Also, ACG showed increased functional connectivity in LBP^18,54^. However, ACG was not found to lead to any change of GMV in PHN patients, and was also hardly found to have significant activity during other BOLD or CBF studies. Our results also showed ACG had more increased MC in LBP. Therefore, ACG might have specific connectivity in LBP patients as compared with PHN.
4. Medial orbital gyrus. In Ung’s study^46^ with 45 LBP patients, left medial orbital gyrus was reported to have decreased GMV than healthy people. However, few studies have mentioned change of medial orbital gyrus in PHN. From the results in this study, we speculated that medial orbital gyrus might be affected much by LBP.
5. SMA. In previous connectivity studies, SMA showed decreased functional connectivity^54^ and nodal efficacy^28^ in LBP, in comparison with healthy controls. SMA also showed decreased MC in LBP compared with PHN. Therefore, SMA, which was repeatedly reported to have been involved in pain modulation, might also have specific connectivity alteration in LBP when compared to PHN.

There were several limitations in our study. First, we lacked healthy subjects with matched age and gender as normal control. Our results could only demonstrate that the brain alterations were the differences between PHN and LBP. However, most previous studies have already showed the aberrant brain structure and activity in pain patients compared with normal control. Besides, using patients as control has the benefits of offsetting negative emotion influence. In further study, we would also enroll normal controls for exposing more specific and comprehensive brain alterations for PNP. Second, we lacked a detailed evaluation scale for emotion, such as the questionnaire for sleep, anxiety and so on. In future study, we would also collect more behavioral data for investigating pain intensity and emotion evaluation. Third, we just investigated the MC in one modality of MRI (T1WI). If multimodal MRI, such as BOLD and DTI, can be acquired, the results might be more persuasive and accurate.

In conclusion, we compared structural characteristics and network features between PHN and LBP by using GMV and MC, respectively. We have obtained reasonable results, which are consistent with previous studies and add new knowledge. For example, ACG might be involved more in LBP modulation, while the network efficacy of medial orbital gyrus might be disrupted more in LBP compared with PHN. Our results showed that, LBP and PHN have different neural mechanisms concerning pain modulation. T1WI-based MC and GMV could be potentially used as neural markers to classify pain disorders.

## Data Availability

All tha data and materials were available at any time.
